# Implementing routine paediatric height/length and weight screening and weight management advice by clinicians: an evaluation

**DOI:** 10.1186/s12913-024-10790-x

**Published:** 2024-03-27

**Authors:** Slavica Krstic, Sarah Dennis, Faye Southcombe, Elizabeth Denney-Wilson

**Affiliations:** 1 Primary and Community Health, South Western Sydney Local Health District, 5 Thomas Rose Drive, Rosemeadow, NSW 2560 Australia; 2 The University of Sydney, Sydney, Australia; 3 Ingham Institute of Applied Medical Research, Sydney, Australia

**Keywords:** Implementation, Weight management, Screening, Evaluation, Overweight, Obesity, Health professional, Children, Adolescents

## Abstract

**Objective:**

To determine the views of health care professionals (HCPs) in South Western Sydney Local Health District (SWSLHD) about the effectiveness of implementation strategies used to increase routine height/length and weight screening, advice, and referral for children and adolescents. A secondary aim was to explore the prevalence of weight bias among HCPs.

**Methods:**

A questionnaire was sent to all HCPs who had undertaken online or face-to-face training between December 2018 and June 2020 in SWSLHD (*n*=840). The questionnaire collected data on their experience of routine height and weight screening and the effectiveness of strategies used in the implementation. It also included a weight bias assessment. Data were provided by the New South Wales (NSW) Ministry of Health on the performance of routine height/length and weight measures entered into the electronic medical records (eMR) in SWSLHD.

**Results:**

Of the 840 questionnaires sent, 87 were undeliverable; of the remaining 753, 285 were returned (38% response rate). More than half (53%, 151/285) of the participants were nurses. Most HCPs agreed that there was a need for routine screening and reported that education, training, and access to resources were the most helpful implementation strategies. Most HCPs were confident in performing routine screening but were less confident in raising the issue of weight with children and their families. Barriers to implementation were lack of time, equipment, appropriate clinical setting, and HCPs’ perceptions and beliefs about obesity.

**Conclusion:**

Routine screening is the first step in identifying children and adolescents at risk of overweight and obesity, but many HCP found it challenging to incorporate into daily practice. Multifaceted strategies are effective in increasing routine screening across diverse healthcare settings so that children and adolescents receive timely and appropriate intervention.

**Supplementary Information:**

The online version contains supplementary material available at 10.1186/s12913-024-10790-x.

## Introduction

Childhood overweight and obesity is a global health problem that is associated with serious adverse health outcomes [1–4]. In New South Wales (NSW), Australia, 24.5% of children and adolescents between 5 and 16 years are overweight or obese [5]. Obesity affects some more than others, and there is evidence of a growing disparity between rates of overweight and obesity among children from low socioeconomic groups [5, 6]. In NSW, South Western Sydney Local Health District (SWSLHD) has both high rates of socioeconomic disadvantage and paediatric overweight and obesity at 37.3% [7].

Healthcare professionals (HCPs) play an essential role in the identification, prevention, and management of overweight and obesity [8, 9]. In NSW, routine screening of child growth is stipulated by policy, which recommends that all children are assessed opportunistically at every interaction with the healthcare system [10]. However, research suggests that HCPs feel ill equipped to identify overweight and obesity or to intervene [11–19]. A lack of time, resources, knowledge, training, and confidence are commonly reported barriers, as well as a lack of referral and treatment options [11, 14, 15, 17, 20–25]. HCPs also report concern about parental receptiveness to discussion around weight and its impact on clinician-patient relationships, and evidence suggests that HCPs engagement is influenced by their weight bias [26, 27]. At odds with this is evidence that children and adolescents and their families find benefit in receiving feedback and advice around weight [28–30].

In 2015, the NSW government identified childhood obesity as a priority action area for NSW health [31, 32]. One strategy was to increase the identification and management of overweight and obesity in clinical services. To support the implementation of this initiative, NSW Health established a performance target of children's height/length and weight measures for all local health districts (LHDs). In 2019, this performance target was set at 70% of children and adolescents (0-16.99 years) attending a healthcare setting should have their growth measured and recorded (height/length and weight) in the electronic medical record (eMR) at least once within the last 90 days [10]. In South Western Sydney Local Health District (SWSLHD), this was operationalised under the Growing Healthy Kids (GHK) action plan [33], which supports HCPs in routinely assessing children’s growth and providing brief interventions and referrals for all children presenting to all SWSLHD healthcare settings, including inpatient, outpatient, oral health, and community settings. This initiative is known as GHK Routine Screening and was further supported by NSW Health’s “Healthy Kids for Professionals” professional education program [31]. This program utilised a 4As approach (assess, advise, assist and arrange) to provide brief interventions targeting obesity prevention and management for children and their families.

Prior to implementing routine GHK screening, no clinical setting in SWSLHD met the Ministry of Health’s required key performance indicators (KPIs) of 70% for height/length and weight screening. Given the high burden of overweight and obesity in SWSLHD, successful implementation of GHK routine screening was critical. However, large HCP program implementation is known to be challenging [11, 34, 35]. To address known barriers, the GHK routine screening implementation utilised multiple strategies to help HCPs embed the 4As approach into routine care. These strategies aimed to actively involve clinicians throughout the implementation process and address barriers to successful implementation (Table 1).

**Table 1:** Suite of strategies used in the implementation of GHK routine screening in SWSLHD

Strategy	Details/purpose of the strategy
Equipment audit and procurement	An audit of weight scales and stadiometers was undertaken across all health care settings. The settings with no equipment or poor quality of equipment were provided with health approved scales and stadiometers.
Weight station set up	Dedicated weight stations were set up (stadiometers, weight scales and resources) were set up across all settings to ensure better/easier access to equipment and resources.
Resources on display	Resources such as posters, referral forms, fact sheets, BMI for age charts, templates for assessment were displayed next to the weight stations to ensure better workflow/visibility and easier access for clinicians.
Education and training of staff	Training included Weight4Kids online (40 minutes) and/or face-to-face (1-2 hours) training to increase staff confidence in height/length and weight measurements, raising issues of weight and referral pathways. Training length varied depending of staff prior knowledge and available training time. Face to face training was offered by GHK coordinator (project officer)
Data audits, reports and feedback	Audit and feedback were provided on monthly to quarterly basis to inform the teams of their progress.
Newsletters	The team with high performance were presented in district newspaper.
Meeting agenda items (individual meetings, team meetings)	Height/length and weight performance was added to teams meeting agendas to ensure regular and frequent discussions to improve focus on growth monitoring and intervention
Action or implementation plans	Teams with consistently low performance or teams with no improvements in their performance were required to complete action plan to ensure clear goals were set for improvement.
Local representative/ Champions	Local representative and Champions were elected to facilitate change within the organisations. Champions were recruited though expression of interest or though managers recommendation.
Local competition	The competition was among paediatric inpatient wards. The ward with best performance was awarded with prize and presented in the district health newspaper for motivation, knowledge sharing and benchmarking

This study aimed to survey HCPs about the effectiveness of these implementation strategies to increase the uptake of routine height/length and weight screening, advice, and referral in SWSLHD. This study also aimed to explore facilitators and barriers to the implementation of routine GHK screening and explore the prevalence of weight bias among HCPs.

## Methods

We conducted a survey of all HCP in SWSLHD who had completed the online and/or face to face training between December 2018 and June 2020. In addition to the survey, we utilised the NSW Health height/length and weight audit, provided quarterly by the Ministry of Health, to compare the achievement of KPIs, which provided us with quantitative data on performance and enabled us to categorise sites. We developed a questionnaire informed by the Consolidated Framework for Implementation Research (CFIR) [36, 37] to ensure that we answered our research question comprehensively. The CFIR is a flexible and commonly used implementation science framework that provides a structural approach to help understand complex factors and processes that may influence implementation. Survey questions were adapted to assess all five CFIR domains (intervention characteristics, inner setting, outer setting, process, and characteristics of individuals) and included closed and open-ended questions [36, 37]. Table 2 provides a short description of the CFIR domains and constructs used in this evaluation.

**Table 2:** CFRI domains – short description and constructs used in this study

Domain	Short Description of domain and constructs used in this study
Characteristics of the intervention	The key component of intervention that may influence implementation success. Two constructs were assessed source of intervention and stakeholders’ perceptions about the relative advantage of implementing the intervention,
Outer setting	The factors or environment that may influence implementation that are external to the setting. Four constructs were assessed: external policy and incentives, patient needs and resources, cosmopolitanism and peer pressure on staff to implement
Inner setting	The factors and characteristics that exist within the setting that may influence implementation. Five constructs were assessed including, structural and cultural characteristic of the setting, communication network within the setting, readiness for implementation and implementation climate
Characteristics of individuals	Individuals’ attributes that may affect implementation. Three constructs were assessed: knowledge and beliefs, self -efficacy and Individual stage of change
Implementation process	Implementation processes that are applied within the service to influence implementation. Two constructs were assessed: such as, evaluation and presence of key stakeholders such as champions.

The survey also included a weight bias assessment: the ‘Fat Phobia Scale’, a validated tool that assesses a person’s attitude to obesity. Items in the scale are graded from 1-5 points with a definition that the lower the scores are, the less bias the person has toward people affected by obesity [38].

The survey was divided into six sections. The first section gathered demographic data from HCPs, including their professions, years of experience, and workplace settings (quantitative) The second section focused on the implementation of the 4 As approach, HCPs' knowledge and beliefs around implementation and the effectiveness of the implementation strategies (Mix of open-ended questions and Likert scales). The third section evaluated the effectiveness of training and the availability of resources. The fourth section focused on routine screening practices and participants' knowledge, confidence, and experience around these processes. The fifth section assessed weight bias among HPCs. The sixth and final section asked HCPs to provide additional feedback on aspects of the program. See supplemental file for survey.

The survey was e-mailed to all eligible HCP. To maximise the response rate, one week prior to the survey, a memo was sent from the District Director of Nursing and Midwifery to all facility managers for distribution to staff. This described the study and encouraged participation by providing a sense of legitimacy [39]. Posters informing HCPs of the survey with survey QR codes were displayed across all facilities. There was also a prize draw to win one of three gift vouchers. Two reminders were sent to HCPs one week apart. As response rates to e-mail surveys are generally low [40], multimode data collection was implemented with electronic and paper-based survey options [41]. The researcher (SK) distributed paper copies via drop-in sessions, team meetings, and meetings with local champions. Participants were also given the opportunity to complete the survey on an iPad. All paper-based surveys were entered into RedCap by a research assistant.

Quantitative data were analysed using IBM SPSS Statistics, Version 27.0. (IBM Corp., Armonk, NY, USA). The responses to closed-ended questions were summarised using frequencies, percentages, means and standard deviations as appropriate. The answers to open-ended questions were coded and categorised using Nvivo-12 software (QSR International). We used a thematic analysis approach [42, 43]. The coding was discussed with two members of the research team (SD, EDW) and revised following discussion. Participants identified the facility in which they worked, and routinely collected data about KPIs for the settings were used to categorise our results. Initially, we aimed to compare settings that successfully achieved the 70% KPI (High implementation sites) with those that did not achieve the KPI (Low implementation sites). However, during the analysis, we discovered significant differences in the implementation between nursing and allied health (AH) staff. This seemed important, so the nursing and allied health staff responses were also analysed separately. As not all strategies were implemented by all sites and not all questions were answered by all participants, the questions with no response or n/a response were excluded from the analysis.

Ethics approval was obtained from the SWSLHD Human Research and Ethics Committee (2019/ETH12871). The results of this study have been published as an abstract and presented as a poster at the International Congress on Obesity 2022 [44].

## Results

### Survey respondents

The survey was sent to 840 HCPs. Of these, 10.3% (*n* = 87) were not delivered due to staff no longer working for the SWSLHD, on leave, or because mail was underivable. Completed surveys were received from 33.9% (*n* = 285) of HCPs, resulting in a response rate of 37.8% (*n* = 285/753). Respondents encompassed a range of clinical disciplines: 53% (*n* = 151) were nurses, 38% (*n* = 108) were allied health (speech pathologists, occupational therapists, dietitians, and psychologists), 6% (*n* = 16) were oral health professionals, including x and x, 2% (*n* = 6) were medical practitioners and 1% (*n* = 4) were aboriginal health workers. Respondents were predominantly HCPs (96%, *n* = 270) and a small number of healthcare executives and senior managers (4%, *n* = 11); four did not indicate their role. When considering healthcare settings, most respondents were from primary and community health settings (40%, *n* = 113), followed by hospital settings (37%, *n*=106), outpatient settings (15%, *n* = 44), and oral health settings (6% *n* = 16) and emergency settings (2%, *n* = 6). More than half of the participants (57%, *n* = 150) worked with children and young people >75% of the time. HCPs with a broad range of experience were represented (Table 3).

**Table 3:** Characteristic of the health professionals participating in the survey

	**Overall**		**High implementation sites**		**Low implementation sites**	
	**n**	**%**	**n**	**%**	**n**	**%**
**What is your healthcare profession? (N 285)**						
Medical	6	2%	5	2%	1	2%
Nursing	151	53%	131	60%	20	31%
Allied Health	108	38%	79	36%	29	45%
Oral Health	16	6%	1	1%	15	23%
Aboriginal Health Worker	4	1%	4	2%	0	0%
**How long have you been working in this profession? (N 285)**						
less than 1 year	13	5%	11	5%	2	3%
1 to 4 years	65	23%	51	23%	14	22%
5 to 9 years	58	20%	47	21%	11	17%
10 to 14 years	52	18%	38	17%	14	22%
15 to 19 years	27	10%	16	7%	11	17%
20 years or greater	70	25%	57	26%	13	20%
**What is your current role in your team? (N 281)**						
Executive/senior management	11	4%	10	5%	1	1%
Clinician	270	96%	206	95%	64	99%
**What setting do you work in most of the time? (N 285)**						
Primary and Community Health	113	40%	113	51%	0	0%
Outpatient	44	15%	0	0%	44	68%
Hospital	106	37%	106	48%	0	0%
Emergency	6	2%	0	0%	6	9%
Oral Health	16	6%	1	1%	15	23%
**Do you still work for SWSLHD? (N 284)**						
Yes	277	98%	213	97	64	99
No	7	2%	6	3%	1	1%
**What proportion of your work is with CYP (2 to 17 years)? (N 281)**						
0-25%	68	24%	46	21%	22	34%
25-50%	27	10%	14	7%	13	20%
50-75%	26	9%	18	8%	8	12%
75-100%	150	57%	138	64%	22	34%

### Survey results

Using constructs from the CFIR, the following sections describe and consider the differences between high and low implementation sites and between AH and nursing staff. The data are presented for each domain of the CFIR and associated questions from the survey (Table 4). Qualitative data from the analysis of the open-ended questions provide further insight (Table 5).

**Table 4:**
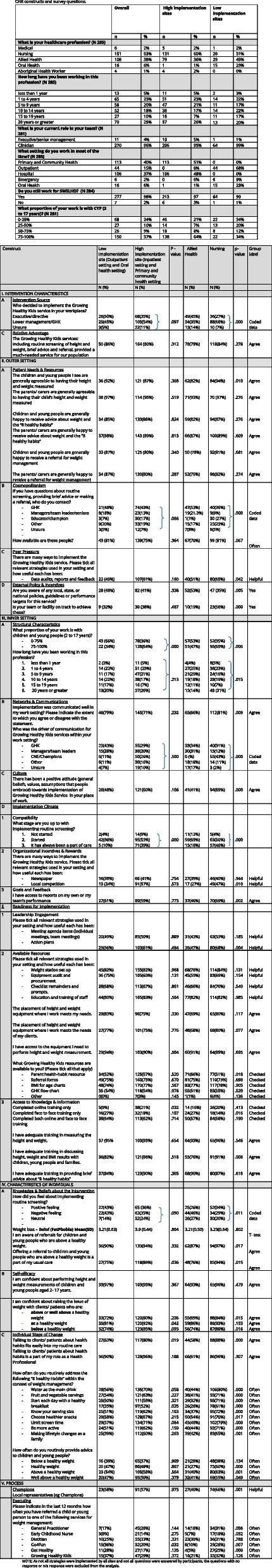
CFIR constructs and survey questions

As not all strategies were implemented by all sites and not all questions were answered by participants, questions with no response or n/a response were excluded from the analysis

**Table 5:** Qualitative data and response examples

Topic	Common teams identified	*Examples of responses by participants*
The need for implementation of GHK strategy	“Bigger picture” (early detection and prevention, education, support for parents, holistic care)	*“Routine screening allows for identification of children below/above a healthy weight, which results in early intervention of nutrition/weight management.”* *“Promote health in children and increase awareness. Detect and prevent poor outcomes.”* *“I find parents appreciate us taking an interest in their child's wellbeing and having options for further follow-up. Otherwise, many parents are unaware of the available options and just feel there is nothing that can be done”* *“ By routine screening it makes parents aware of their child’s height and weight and also their BMI”* *“We are empowering the parents/ patients with knowledge when we educate and refer their child”*
	high prevalence of childhood overweight and obesity	*“We have an increasing number of the paediatric patients that are admitted to the ward and are above a healthy weight.”* “*High population of disadvantaged/vulnerable families to poor access to health promotion and services. High rates of overweight clients”* *“With the growing number of obese children, I think this service has been much needed and we'll embraced”*
	Core business	*“Continuation of what we have been assessing weight & BMI & discussion with parents* *“ We were already providing this within Child and Family health - but it's nice having a clear pathway and seeing other services performing this so we can get a more comprehensive picture”*
Barriers to implementation	Too busy, not enough time	*“Many staff feel overwhelmed with addition of this task on top of our already high demand, fast based environment.”* *“Expected to implement without the additional space or staff into existing clinic time which is already at capacity.”* *“It adds extra consultation time to an already stretched workforce”.* *“Really inconvenient and time consuming tasks to complete in a time poor environment”*
	Won’t help/weight bias	*Agree families need the service, but when provided the service, they often do not want the service thus not making it effective.”*
	Out of scope/not my role	*“I can see how it may be appropriate in a situation such as a doctor, nurse, dietitian or nutritionist taking these measurements; however, it is outside the scope of my professional role.”* *We are consistently being required to add more and more tasks to our day to day operations and this was not something that as Speech Pathologists we believed was essential to our role*
	Not appropriate setting	*It takes time out of already short sessions on an area that is not my clinical field of expertise and in an area that can be quite sensitive”* *“*Agreed that it is important, but in the outpatient plaster clinic setting it seems out of place” “We are not the first encounters for the children that need further help. These people are normally the family GP's childcare worker, teachers”
	Negative impact on relationship	*“While this is a worthwhile service, there are concerns around the appropriateness of allied health clinician to provide feedback on this to families and the risk of affecting engagement with families.”* “*Concern exists around how the physical measuring of children's weight by psychologists will impact on the therapy space feeling safe, non-judgemental and client goal directed”* “The counselling staff would not get involved in weighing their clients - doing so blurs the boundaries in the therapy space and can affect the sense of trust and safety that clients feel with their counsellor”
	No equipment, not appropriate location/space	*Staff find it difficult to complete patients’ height and weight due to inadequate equipment”* *“Poor location of scales & measuring devices”* *“Measuring equipment not readily available and not appropriate for age groups”*
Training	Most useful	*“Face to face components as staff Q & A with trainer relating to the implementation in specific local services is helpful.”* *“Both online and Face to Face. Online allows you to read up on things and Face to Face brings it all together”*
	Least useful	*Lengthily online module, time consuming”* *‘due to overall volume of online training staff have to complete the addition of extra and multiple module is difficult to prioritise in amongst a clinicians busy schedule*”.
	Training missing	*“It can be a very challenging discussion with parents and can be a very emotional time for parents. It would be good to have training on this in particular”* *“Ongoing update on patient progress”* *“Further training requires re having discussion with families”* *“How to carry out effective and culturally sensitive discussions about healthy weight with patients and parents/carers and especially adolescents”* *“Would of like to do more role play”* *“More strategies to engage parents who are not interested of don't think their child weight/BMI is an issue,”*
Improvements in implementation	Equipment and better space	“*Placing equipment in appropriate area to weigh and measure children respectfully eg. Not in the common area where patient sit for allied health, mental health, drug and alcohol services etc.”* *“Having it in a more private place (ie. Not in the waiting room where everyone can see)”* *“Having more equipment in each consult room”* *“Having a height and weight machine in isolation rooms for ?COVID patients as the results can take up to 24 hours and then therefore delay the measurements.”*
	Flagging system	*“Electronic remainder on client records- shared accountability to help clinicians keep a truck of when new measurements are required”* *“Reminding staff at meetings regularly to screen.”* *“To have an alert system on Powerchart to alert clinician when BMI/weight is above healthy weight for a patient once height and weight are entered.*
	Extra staff and extra time	“*Having an extra person to do the screening as it takes a lot of time”* *“Additional time allocated for the appointment”*

#### Domain: Intervention characteristics

The implementation of routine GHK screening was deemed necessary by most HCPs across both high (80%, *n* = 164) and low implementation sites (86%, *n* = 50). Compared to AH, nurses were more likely to agree with this statement, with 84% (*n* = 118) of nurses expressing support compared to 79% (*n* = 78) of AH. A greater proportion of HCPs in high implementation sites and nurses perceived the policy to be locally initiated by GHK representatives and lower management. In contrast, low implementation sites and AH perceived the source to be from higher management and external sources (directors, executives, and NSW Health) (Table 4). Qualitative responses suggested that the service was an important opportunity for preventive healthcare, and the mechanism for this may be through early detection and education and increased awareness of the high prevalence of childhood overweight and obesity (Table 5).

HCPs identified a lack of available time as the primary barrier to implementation. The second most noted barrier, particularly among AH staff, was concerns about scope of practice. Other barriers commonly reported included a belief that obesity was treatment resistant and a concern for the suitability of the clinical setting for assessing children’s height and weight. Some participants also reported concerns about the impact of raising the issue of weight on the therapeutic relationship (Table 5).

#### Domain: outer setting

Three characteristics within the outer setting appeared to have influenced implementation: patient engagement, performance data reporting and HCP awareness. Generally, HCPs felt that children, adolescents, and their carers were agreeable to growth assessment, advice, and referral. This did not differ by level of implementation. There was a nonsignificant difference in communicating the performance data, with high implementation sites (61%, *n* = 107) being more likely to find this strategy helpful than low implementation sites (49%, *n* = 22); *p* = 0.160. HCP awareness of the performance data was significantly greater among AH (53%, *n* = 52) compared to nurses (35%, *n* = 47), *p* = 0.005. Despite this, only 19% (*n* =10) of AH agreed that their team was on track to achieve the performance target compared to 56% (*n* = 25) of nurses (*p* = 0.000).

#### Domain: inner setting

Across high implementation sites, a greater proportion (64%, *n* = 138, *p* = <0.001) of HCP reported a predominantly paediatric workload. This was also evident when comparing professions, with only 47% (*n*=51) of AH reporting working with children more than 75% of the time compared to 65% (*n* = 95, *p* = 0.006) of nurses. Participants believed that communication was effective. However, nurses reported better communication (81%, *n*=112) than AH (66%, *n*=65) (*p*=0.009). Across high implementation sites, communication was driven by local clinical nurse educators/champions, while across low implementation sites, communication was predominantly from external sources. AH reported lower rates of positive attitudes associated with implementation compared to nursing (41%, *n* = 41 vs 69%, *n* = 94, respectively, *p* = <0.001). High implementation sites were more likely (39%, *n* = 71) to report that height and weight screening was always a part of routine care compared to only 10% (*n* = 5, *p* = <0.001) in low implementation sites. Nurses were also more likely to report that this was part of usual care than AH (46%, *n* = 57 vs 18%, n= 15, *p* = <0.001).

The strategies that were found to be the least helpful were newspapers and local competition. AH staff reported having less access to feedback on their performance than nursing staff (46%, *n* = 37 vs 69%, *n* = 70, *p* = 0.002). Including performance data as a meeting agenda item was a useful strategy for half of the participants. Nurses found using action plans more helpful than AH (*p* = 0.004). Education and training were frequently identified as helpful strategies across both sites. Most HCPs reported adequate training in measuring, discussing height and weight and providing brief advice. This did not vary across the sites; however, nurses were more likely to report adequate training (91% *n*=65) than AH (76%, *n*=55) (*p*= 0.008). Most HCPs completed online and face-to-face training but found the face-to-face training most useful.

Weight station setups, equipment audits and procurement were found to be useful. Both sites reported high access to adequate equipment for height and weight measurements. However, access to resources such as healthy habits resources, referral forms, BMI for age charts, and GHK referral flow charts were reported less frequently. Additionally, qualitative data revealed that HCPs reported that implementation would be improved by better equipment location to aide access and privacy, alerts in the electronic medical system and more staff and greater time allocation (Table 5).

#### Domain: characteristics of individuals

The mean score on the Fat Phobia scale (3.2, SD) did not vary significantly across the sites (*p*= 0.804) or among AH and nursing (*p*=0.802). However, it suggests a moderate level of weight bias among staff. The participants described various feelings about implementing routine screening. Low implementation sites and AH clinicians were more likely to have negative feelings, mainly feeling ‘*stressed*’. Conversely, high implementation sites were more likely to use positive words such as ‘excitement’. Nurses were more neutral in their language, as they felt this was part of their usual practice. Most HCPs reported that discussing weight did not always fit into their usual practice. There was a significant difference across the low and high implementation sites (63%, *n* = 27 vs 80%, *n* = 117, *p* = 0.019) as well as between AH and nursing (58%, *n*= 44 vs 88%, *n*= 88, *p* = 0.000). Most clinicians (93-97%) were confident in performing height and weight measurements but were less confident in raising the issue of weight with families, especially with those outside a healthy weight range. Nurses were more confident in raising the issue and more likely to provide advice than AH. Advice was most frequently offered to parents of children above a healthy weight. The strategy most discussed was ‘water as the main drink’. Referral of children and adolescents was regarded as part of usual care (>75%) in high implementation sites and by nurses, with a high awareness of local referral services reported by all HCPs.

#### Domain: process

Overall, more than half of all participants found having a local champion helpful, significantly more so among nursing compared to AH (66%, *n* = 74 vs 40%, *n* = 27, *p* = 0.001)). Low rates of referrals were reported across all sites; however, nurses were more likely to make referrals compared to AH. The total number of strategies used across the sites varied (Table 6). Overall, low implementation sites and AH used the least number of strategies when compared to high implementation sites and nursing.

**Table 6:** Percentage of intervention strategies NOT used across the sites and health professionals

	**Low implementation**	**High implementation**	***P* value**	**Allied Health**	**Nursing**	***P* value**
	**N (%)**	**N(%)**		**N(%)**	**N(%)**	
Equipment audit and procurement	7(13%)	27(14%)	.810	20(21%)	11(8%)	.007
Weight station set up	1(2%)	9(5%)	.351	7(7%)	3(2%)	.057
Resources on display	1(2%)	7(4%)	.531	5(5%)	3(2%)	.209
Education and training of staff	-	4(2%)	.293	2(2%)	2(1%)	.696
Data audits, reports and feedback	9(17%)	22(11%)	.265	17(18%)	14(10%)	.098
Newsletters	12(22%)	35(18%)	.457	27(28%)	19(14%)	.008
Meeting agenda items (individual meetings, team meetings)	13(24%)	28(14%)	.080	24(25%)	17(13%)	.014
Action or implementation plans	9(17%)	30(15%)	.785	21(22%)	18(13%)	.083
Local representatives (e.g. Champions)	15(27%)	40(20%)	.253	29(30%)	26(19%)	.044
Local competition	16(30%)	49(25%)	.480	33(34%)	31(23%)	.056

## Discussion

This study reports on a relationship between implementation strategies and implementation performance among HCPs implementing routine growth assessments of children and adolescents. High implementation sites were those that had a focus on paediatric patients, a local champion, and staff, particularly nurses, who regarded routine height and weight measurement as part of their usual clinical role and scope of practice. This study highlighted that the construct related to GHK program attributes (intervention characteristics domain) seemed to be a key feature for successful implementation. Implementation was enhanced by staff support and understanding the “bigger picture” of the health problem. In our study, most HCPs, after attending GHK training sessions, understood the importance of routine screening and their role in the prevention and management of childhood obesity. This was an important finding, as without acknowledging their role in the overall obesity strategy, the implementation may not have succeeded. Research has shown that clinicians are known to be more likely to embrace patient safety initiatives when they clearly understand the evidence supporting the practice [45–47].

Other key factors that aided the implementation process were within the inner setting domain of CFIR and included providing education, equipment and resources to staff. Both online and face-to-face education sessions were valued. However, it seemed that having face-to-face training with the opportunity to practice the skills using role play and asking questions, particularly focusing on difficult conversations, was preferred. This was evident in a randomised control trial that compared two training methods for addressing obesity screening and intervention: interactive live training and web-based self-study training. Both methods were effective, but live training was more acceptable [48]. Similarly, other studies found that conventional approaches such as conferences and lectures have been shown to be ineffective in implementing guidelines, while interactive techniques such as workshops and practical sessions with evaluations have consistently yielded positive results in 10 out of 11 systematic reviews [34].

Although providing education on paediatric obesity screening and management for HCPs is effective [48, 49], it is not enough to induce clinical practice changes on its own [45]. Research has shown that combining education with the influence of opinion leaders can be more effective [45]. In our study, local coordinators, champions, and educators played a crucial role in implementing changes. Certain individuals, known as “key drivers” or “champions,” are important in bringing about change within an organisation [50, 51]. They offer motivation, encouragement and work hard to acquire necessary resources and support for the team’s success [47]. They also maintain positive relationships with other HCPs. They share information, promote the adoption of new innovations, and demonstrate and train staff on the new initiative [45]. This benefit of the coordinator and champion roles in this implementation should not be underestimated.

Providing staff with equipment and weight stations to assist staff in having weight conversations with patients and families was reported to be valuable. However, HCPs reported the need for weight stations to be placed in private areas to ensure confidentiality, and this was not always possible. These steps were critical for successful implementation, as a lack of resources and equipment has been reported as a barrier to implementation [11, 52]. Additionally, a lack of privacy can make it difficult for HCPs to communicate openly and honestly with families, which can impact children’s care.

In the ‘outer setting’ domain, we identified sites where awareness of the policies did not influence implementation, with settings with low awareness reporting higher KPIs. Just being aware of the policy and practices does not result in behaviour change, and there may be a lack of necessary support to effectively implement the policy. We also discovered that communication played a vital role in implementing changes. Our findings showed that at successful implementation sites, communication was driven by internal sources (local clinical nurse educators, champions, or coordinators), while external communication sources (directors, managers, policy directives) were more prominent at less successful sites. Top-down implementation strategies and unrealistic goals can reduce motivation, but involving healthcare professionals in the implementation process from the bottom up can better sustain motivation [53].

In this project, most HCPs found audit and feedback to be a helpful implementation strategy. This aligns with similar findings in other studies [45, 54, 55] and highlights the importance of settings and monitoring KPIs when implementing new change. Our study found that sites with successful implementation (met KPIs) utilised multiple implementation strategies. This aligns with the literature emphasising the importance of using multiple strategies instead of relying on a single strategy [11, 34, 56, 57].

Within the CFIR framework, the domain of ‘Inner Setting’ highlights the significance of structural characteristics of the implementation sites in shaping the success of routine screening. This is supported by similar findings in other research studies [45, 51]. In our findings, hospitals and community centres were more receptive to implementing changes compared to oral health and outpatient settings, likely due to their higher proportion of paediatric patients (over 70%) and a greater proportion of nursing staff. These settings integrated changes into their daily routine, unlike settings with mainly adult patients, where staff may only have performed tasks with paediatric patients a few times a week.

Within the “characteristics of individuals’ domains”, we observed that potential HCPs’ beliefs and attitudes negatively impacted implementation and overall clinical care, affecting patients directly. HCPs’ belief that the intervention was not part of their role was commonly described as a barrier to implementation [50]. In our study, some HCPs expressed the belief that weight screening was not their responsibility and was outside their scope of practice, hindering the implementation process. This was particularly prevalent among AH staff, despite the literature suggesting their effectiveness in promoting healthy behaviours in children [49]. Additionally, some HCPs in our study believed their setting was not suitable for screening due to a focus on other clinical issues. Some HCPs felt uncomfortable discussing weight issues with parents, fearing it may damage their relationship. An individual is more likely to resist the change if there is a negative belief about the usefulness of the intervention or a belief that the intervention is not part of one’s role [50]. It is important to address these barriers and negative beliefs to promote positive change.

The culture of the setting also impacts the success of implementation [58]. Our findings revealed that sites that reported more positive attitudes toward the implementation, as reflected in their general beliefs, values, and assumptions, performed better. In line with our research, a study examining ways to enhance chronic illness care emphasizing the significance of cultivating a positive team culture and enlisting team champions to implement changes effectively [47].

This study highlighted that HCPs still exhibit weight bias, despite receiving education and training and having an awareness of weight issues. Weight bias could affect GHK implementation, as clinicians who have weight bias may spend less time with patients, be reluctant to perform screening and perform fewer interventions [59–61]. This can affect patients’ wellbeing and future health choices [62, 63], indicating the need to further tackle weight bias. The presence of weight bias in healthcare settings should be a matter of concern for policymakers and should be addressed urgently. While there is limited published research on interventions aimed at reducing weight bias, a recent systematic review by Moore (2022) [64] found that face-to-face or online education sessions can result in small to moderate reductions in weight bias. However, it is important to note that most of these studies have focused on healthcare students, and there is still a lack of research on interventions for established HCPs with set beliefs.

Practical barriers, such as lack of time and heavy workload, were also reported as common barriers, consistent with previous literature [11, 35, 52, 65]. Of note is the finding from our study that while most HCPs were confident in performing height and weight measurements, they had varying levels of confidence when discussing weight with families, particularly with individuals who were well above a healthy weight (obese category). There is a need for additional training to ensure that these conversations are handled in a sensitive and helpful way.

Our project successfully addressed the main barriers, such as clinicians’ lack of knowledge, skills, equipment and resources. However, there were areas that needed improvement based on feedback from HCPs. HCPs need continuous training and refresher courses, role-playing difficult conversations, especially among adolescents. HCPs also stated that evaluating patients’ outcomes would also be beneficial and recommended better equipment and better weight station position to allow for privacy and having electronic prompts to serve as a reminder to perform height/length and weight measurements.

Overall, our study found that some CFIR constructs manifested differently across the sites and HCPs, emphasising the need for a combination of organisational strategies and contextualised structures to establish routine screening practices. Therefore, it is important to recognise that a single implementation plan may not work the same way for all HCPs.

### Limitations

This study has some limitations. Firstly, the response rate was disappointing and the number of respondents per profession varied which affects the generalisability of our findings. This is despite the efforts to maximise the response rate by providing participants with various options to complete the questionnaires, such as mail, e-mail and paper versions collected from workplaces. Second, this study did not explore relative priority: number of initiatives or changes rolled out recently across each facility and whether this impacted implementation. Third, this study did not explore leadership engagement, such as the commitment, involvement, and accountability of leaders and managers, as this may have influenced the success of implementation. While this is a cross-sectional study and we cannot imply causality, those providers who reported higher implementation also reported specific uses of, or awareness of, strategies suggesting that they might have had a role in clinician behaviour change. We acknowledge that there may be other unmeasured variables affecting both of these.

### Policy implications

To facilitate successful implementation of complex interventions, the choice of implementation strategies needs to be based on an understanding of the barriers relevant to the setting (context) in which the implementation occurs. Multiple strategies should be offered with a key coordinator and champions to lead the change. Provision of education to staff and providing the staff with equipment and resources with regular feedback are crucial in improving routine screening. A single training session is unlikely to lead to successful implementation, and continuous training/refreshers are needed. Training should focus on having difficult conversation and should include case studies focusing on program evaluation. Addressing weight bias is crucial to successfully implement the 4As approach in the clinical setting. It may be advisable to focus implementation efforts in paediatric settings only.

## Conclusion

Screening for obesity of all children and young people presenting to health care facilities, combined with providing advice and referral to appropriate intervention services, are all important strategies to prevent and reduce obesity. Many healthcare professionals understand the importance of routine screening and referrals but do not adopt this practice. This evaluation demonstrated that to achieve more efficient and effective implementation of routine screening practices in any setting, multifactorial strategies are needed, and barriers such as HCP knowledge, equipment, lack of time and space, and HCP perceptions/beliefs must be addressed. The findings of this study may inform further strategies for other settings and contexts.

### Supplementary Information

**Supplementary Material 1.** 

## Data Availability

The data used to generate the results in the paper is not available.
