# Supplementary material for: Implementing routine paediatric height/length and weight screening and weight management advice by clinicians: an evaluation
Source: BMC Health Serv Res. 2024 Mar 27;24:380. doi: 10.1186/s12913-024-10790-x (PMC10967040; doi:10.1186/s12913-024-10790-x)
Supplement: Supplementary file 1 — Supplementary Material 1. [file 12913_2024_10790_MOESM1_ESM.pdf]

The Growing Healthy Kids Study: Evaluation Survey

The Growing Healthy Kids Service includes three strategies:

- *Measuring children and young people; known as routine screening of height and weight*
- *Providing brief advice; this includes discussing height, weight and BMI results with children, young people and their carers, and providing healthy lifestyle advice.*
- *Referring children and young people who are above a healthy weight to appropriate services*

We are interested in hearing your thoughts on these strategies, and what it has been like implementing them in your setting. Some questions will be about your team environment and some about your experience as a clinician.

Please complete the surveys below
Thank you!

BACKGROUND

What is your healthcare profession?

- ○ Medical
- ○ Nursing
- ○ Allied Health
- ○ Oral Health
- ○ Other, please specify ____________________

How long have you been working in this profession?

- ○ Less than 1 year
- ○ 1-4 years
- ○ 5-9 years
- ○ 10-14 years
- ○ 15-19years
- ○ 20 years or longer

What is your current role in your team?

- Director
- Manager
- Team Leader
- Educator
- Clinician
- Senior Clinician
- Nurse
- Medical officer
- Dental officer
- Dental therapist
- Dental Assistant
- Other please specify ____________________

What setting do you work in most of the time?

- Primary and Community Health
- Outpatient
- Hospital
- Oral Health
- Mental Health
- Emergency
- Other, please specify___________________

Do you still work for SWSLHD?

- Yes
- No

What proportion of your work is with children and young people (2 to 17 years)?

- 0-25%
- 25-50%
- 50-75%
- 75-100%

GENERAL: The Growing Healthy Kids Service

Who decided to implement the Growing Healthy Kids service in your workplace?

Implementation was communicated well in my work setting.

Please indicate the extent to which you agree or disagree with this statement.

- ○ Strongly disagree
- ○ Disagree
- ○ Neither agree nor disagree
- ○ Agree
- ○ Strongly agree

Who was the driver of communication for Growing Healthy Kids services within your work setting?

The Growing Healthy Kids services (routine screening of height and weight, brief advice and referral) provides a much needed service for our population. Please indicate the extent to which you agree or disagree with this statement.

- ○ Strongly disagree
- ○ Disagree
- ○ Neither agree nor disagree
- ○ Agree
- ○ Strongly agree

Briefly explain why you think this

There has been a positive attitude (general beliefs, values, assumptions that people embrace) towards the implementation of Growing Healthy Kids Service in your workplace. Please indicate the extent to which you agree or disagree with this statement.

- ○ Strongly disagree
- ○ Disagree
- ○ Neither agree nor disagree
- ○ Agree
- ○ Strongly agree

Briefly describe an example that highlights this:

Are you aware of any local, state, or national policies, guidelines or performance targets for this service?

- ○ Yes; Please specify_______________________________________
- ○ No

Is your team or facility on track to achieve these?

- ○ Yes
- ○ No
- ○ Unsure

There are many ways to implement the Growing Healthy Kids service. Please tick all relevant strategies used in your setting and how useful each has been.

Strategy	Unhelpful	Somewhat helpful	Neutral	Helpful	Very helpful	N/A
Equipment audit and procurement						
Weight station set up						
Resources on display						
Education and training of staff						
Data audits, reports and feedback						
Newsletters						
Meeting agenda items (individual meetings, team meetings)						
Action or implementation plans						
Local representatives (e.g. Champions)						
Local competition						
Checklist or prompt reminders						

What other strategies have you used?

TRAINING

Please indicate all the training have you received for the Growing Healthy Kids Service? (Tick all that apply)

- Weight4KIDS Online Training
- Face to Face (2 hour session)
- Face to Face (1 hours session)
- Face to Face (unsure of length)
- Other, please specify
  ____

What aspects/components of the training have been **MOST** useful?

What aspects/components of the training have been **LEAST** useful?

What training is missing?

What Growing Healthy Kids resources are available to you? (Please tick all that apply)

- Parent health-habit resources
- Referral forms (Go4Fun, Get Healthy, Growing Healthy Kids Service referral, GP)
- BMI for age charts
- Growing Healthy Kids Service process flow-chart
- Other, please specify____

If you have questions about routine screening, providing brief advice or making a referrals, who do you contact?

- How available are these people?
  - Never
  - Rarely
  - Sometimes
  - Often
  - Always

**ROUTINE SCREENING**
*Routine screening refers to measuring the height and weight of children and young people aged 2 to 17 years. The Ministry of Health recommend that all Health Care Professionals should measure the height and weight of **all** children and young people as a part of high quality routine care.*

What stage are you up to with implementing routine screening?

- Not started yet
- We are thinking about it
- We are getting ready to start
- We just started
- We have been doing it for a while
- It has always been a part of our routine care
- Unsure

How did you feel about implementing routine screening? Excited? Stressed? Enthusiastic?

Please indicate the extent to which you agree or disagree with the following statements.

	Strongly Disagree	Disagree	Neutral	Agree	Strongly Agree	N/A
I have access to reports on my own or my team's performance in routine screening						
I have adequate training in measuring the height and weight of children and young people						
I am confident about performing height and weight measurements of children and young people aged 2- 17 years						
I have access to the equipment I need to perform height and weight measurements						
The placement of height and weight equipment where I work meets my needs						
The placement of height and weight equipment where I work meets the needs of my clients						
The *children and young people* I see are generally agreeable to having their height and weight measured						
The *parents/ carers* are generally agreeable to having their child's height and weight measured						

BRIEF ADVICE

*Providing brief advice includes discussing height, weight and BMI results with children, young people and their carers, and providing healthy lifestyle advice. The Ministry of Health recommends that all Health Care Practitioners provide brief advice about weight and healthy habits to **all** children and young people aged 2 to 17 years.*

Please indicate how often you routinely address the following "8 healthy habits" within the context of weight management. If you don't routinely use an element of this strategy then select n/a.

	Never	Rarely	Sometimes	Often	Always	n/a
Water as the main drink						
Fruit and vegetable servings						
Start each day with a healthy breakfast						
Know your serving size						
Choose healthier snacks						
Limit screen time						
Be more active						
Get enough sleep						
Making lifestyle changes as a family						

How often do you routinely provide advice to children and young people who are:

	Never	Rarely	Sometimes	Often	Always	N/A
Below a healthy weight						
Healthy weight						
Above a healthy weight						
Well above a healthy weight						

Please indicate the extent to which you agree or disagree with the following statements.

	Strongly Disagree	Disagree	Neutral	Agree	Strongly Agree	N/A
I have adequate training in discussing height, weight and BMI results with children, young people and families						
I have adequate training in providing brief advice about "8 healthy habits"						
I am confident about raising the issue of weight with clients/ patients who are **above or well above** a healthy weight						
I am confident about raising the issue of weight with clients/ patients who are at a **healthy** weight						
I am confident about raising the issue of weight with clients/ patients who are **below** a healthy weight						
Talking to clients/ patients about health habits fits easily into my routine care						
Talking to clients/ patients about health habits is a part of my role as a Health Professional						
Children and young people are generally happy to receive advice about weight and the "8 healthy habits"						
The *parents/ carers* are generally happy to receive advice about weight and the "8 healthy habits"						

REFERRAL

Treating overweight and obesity in children and young people is important. Children and young people who are outside the healthy weight range may require referral to appropriate services.

Please indicate the extent to which you agree or disagree with the following statements.

	Strongly Disagree	Disagree	Neutral	Agree	Strongly Agree	N/A
Offering a referral to children and young people who are above a healthy weight is a part of my usual care.						
I am aware of local services and referral options for children and young people who are above a healthy weight.						
I routinely offer referrals for children and young people who are above a healthy weight						
Children and young people are generally happy to receive a referral for weight management						
The *parents/ carers* are generally happy to receive a referral for weight management						

Please indicate in the las 12 months how often you have referred a child or young person to one of the following services for weight management.

	Never	Rarely	Sometimes	Often	Always	n/a
General Practitioner						
Early Childhood Nurse						
Dietitian						
Go4Fun						
Get Healthy						
Growing Healthy Kids						
Children's Hospital Westmead weight clinic						

THOUGHTS AND BELIEFS

Do you consider yourself to be overweight or obese?

- ○ Yes
- ○ No
- ○ Unsure
- ○ Would rather not say

Listed below are 14 pairs of adjectives sometimes used to describe obese or fat people. For each adjective pair, please place an X on the line closest to the adjective that you feel best describes your feelings and beliefs.

1.	Lazy	________	________	________	________	________	Industrious
		5	4	3	2	1	
2.	No will power	________	________	________	________	________	Has will power
		5	4	3	2	1	
3.	Attractive	________	________	________	________	________	Unattractive
		5	4	3	2	1	
4.	Good self-control	________	________	________	________	________	Poor self-control
		5	4	3	2	1	
5.	Fast	________	________	________	________	________	Slow
		5	4	3	2	1	
6.	Having endurance	________	________	________	________	________	Having no endurance
		5	4	3	2	1	
7.	Active	________	________	________	________	________	Inactive
		5	4	3	2	1	
8.	Weak	________	________	________	________	________	Strong
		5	4	3	2	1	
9.	Self-indulgent	________	________	________	________	________	Self-sacrificing
		5	4	3	2	1	
10.	Dislikes food	________	________	________	________	________	Likes food
		5	4	3	2	1	
11.	Shapeless	________	________	________	________	________	Shapely
		5	4	3	2	1	
12.	Under eats	________	________	________	________	________	Overeats
		5	4	3	2	1	
13.	Insecure	________	________	________	________	________	Secure
		5	4	3	2	1	
14.	Low self-esteem	________	________	________	________	________	High self-esteem
		5	4	3	2	1	

FEEDBACK

In your work setting, what would make the GHK service better?

If we need more detailed information, can we contact you in the future?

- If so, please provide your name and contact details?

Thank you for taking the survey

Please ensure you have signed Participant Information Consent form.

Have a nice day!
